# Computer-Assisted Navigation in Shoulder Arthroplasty: A Narrative Review

**DOI:** 10.3390/jcm14082763

**Published:** 2025-04-17

**Authors:** Marina Marescalchi, Alessandro El Motassime, Luca Andriollo, Alberto Polizzi, Giuseppe Niccoli, Vincenzo Morea

**Affiliations:** 1 Ortopedia e Traumatologia, Fondazione Poliambulanza, 25124 Brescia, Italy; 2 Dipartimento di Scienze geriatriche e ortopediche, Università Cattolica del Sacro Cuore, 00168 Rome, Italy; 3 Department of Orthopedics, Ageing and Rheumatological Sciences, Fondazione Policlinico Universitario A. Gemelli IRCCS, 00168 Rome, Italy; 4 Artificial Intelligence Center, Alma Mater Europaea University, 1090 Vienna, Austria

**Keywords:** computer-assisted navigation, reverse shoulder arthroplasty, GPS navigation, shoulder replacement, shoulder osteoarthritis

## Abstract

Shoulder arthroplasty, including total shoulder arthroplasty (TSA) and reverse shoulder arthroplasty (RSA), is a well-established procedure for treating degenerative, post-traumatic, and inflammatory conditions of the shoulder joint. The success of these surgeries depends largely on the precise placement of implants, which helps restore proper joint mechanics, reduce complications, and extend the lifespan of the prosthesis. However, achieving accurate implant positioning can be challenging, especially in cases involving severe bone loss, anatomical deformities, or prior surgeries. Poor alignment can lead to instability, implant loosening, and the need for revision surgery. Computer-assisted navigation has become an important tool in shoulder arthroplasty, providing real-time intraoperative guidance to improve surgical accuracy and consistency. By integrating preoperative 3D imaging with intraoperative tracking, navigation technology allows surgeons to optimize glenoid component placement, reducing the risk of malalignment and mechanical failure. Research suggests that navigation-assisted techniques improve precision, enhance functional outcomes, and may even reduce complication rates by optimizing fixation strategies, such as using fewer but longer screws in RSA. Despite its benefits, navigation in shoulder arthroplasty is not without challenges. It requires additional surgical time, increases costs, and demands a learning curve for surgeons. However, with advancements in artificial intelligence, augmented reality, and robotic-assisted surgery, navigation is expected to become even more effective and accessible. This review explores the current impact of navigation on clinical outcomes, its role in complex cases, and the future potential of this technology. While early results are promising, further long-term studies are needed to fully assess its value and establish best practices for its routine use in shoulder arthroplasty.

## 1. Introduction

Shoulder arthroplasty has experienced significant evolution, influenced by advancements in implant design, surgical techniques, and perioperative management strategies since the 1950s. This evolution can be traced back to Charles Neer’s design and implantation of the first shoulder hemiarthroplasty, followed by the introduction of Neer’s total shoulder arthroplasty in 1974. Subsequently, reverse designs were proposed by Kolbel and Friedebold, Reeves, and Fenlin in the 1970s, culminating in the emergence of the Grammont design and its Delta III version [1,2,3,4,5].

**Figure 1:**
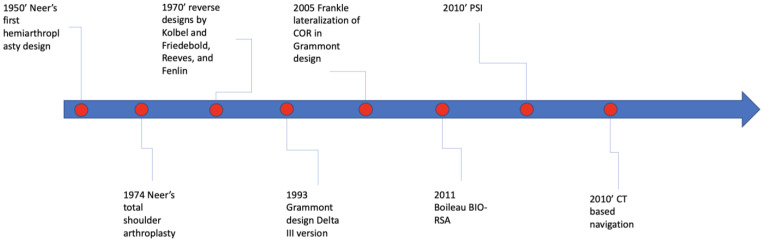
Timeline of the main steps in shoulder arthroplasty [COR: center of rotation; BIO-RSA: Bony-Increased Offset Reverse Shoulder Arthroplasty; PSI: patient-specific instrumentation; CT: computed tomography].

In 2005, Frankle et al. sought to expand upon the foundational Grammont-style RSA constructs [6]. Accordingly, a novel RSA prosthesis was developed featuring a more laterally positioned center of rotation (COR), with the aim of closely reproducing the anatomical center of rotation of the native humerus. In 2011, Boileau et al. introduced the Bony-Increased Offset Reverse Shoulder Arthroplasty (BIO-RSA), specifically in the context of posterior glenoid deficiencies [7]. The rise of patient-specific instrumentation (PSI) aligned with improvements in high-resolution CT imaging and 3D planning software, allowing for the creation of individualized bone models and customized surgical jigs. These tools help surgeons prepare the glenoid and insert implants based on a patient-specific plan, marking progress in personalized orthopedic surgery. Preliminary studies show that PSI enhances glenoid component positioning accuracy compared to standard instrumentation [8,9]. Recently, Guided Personalized Surgery (GPS) platforms have been developed for reverse shoulder arthroplasty, inspired by navigation successes in total hip and knee replacements (Figure 1).

One of the most prevalent indications for this procedure is osteoarthritis (OA), a progressive degenerative disorder characterized by chondral degradation, osteophyte formation, and joint space narrowing, ultimately leading to pain and functional impairment. Total shoulder arthroplasty (TSA) and reverse shoulder arthroplasty (RSA) have been widely adopted to restore joint function and alleviate symptoms in patients with advanced OA; however, ensuring accurate implant positioning remains a major challenge, particularly in cases of severe bone loss and complex anatomical variations.

A growing number of younger patients are receiving prosthetic replacement as a result of traumatic, rheumatic, or drug-induced conditions [10,11]. Therefore, it is imperative that implants provide optimal durability, precision, and consistency to enhance long-term clinical outcomes.

Additionally, factors such as patient-specific anatomy, previous surgical interventions, and varying degrees of joint degeneration introduce further complexities in achieving optimal outcomes. Inadequate component positioning can lead to mechanical complications, reduced implant longevity, and a higher likelihood of revision surgery [12,13,14]. The 2024 Australian Orthopaedic Association National Joint Replacement Registry reports a 10-year revision rate of 7.5% for total stemmed anatomic shoulder arthroplasty and 5.5% for total stemmed reverse arthroplasty [15].

At the five-year mark, the cumulative revision rates stand at 8.1% for anatomic TSA and 4.6% for reverse TSA. The primary reasons for these revisions include instability and loosening, which represent 38.5% and 18.0% of the cases, respectively [16]. Failure of the glenoid component is identified as the main cause of long-term clinical failure in both anatomic and reverse TSA. Additionally, the accurate positioning of the glenoid component is a vital factor that affects the procedure’s success and the prevention of postoperative instability [2,17,18,19].

Given these challenges, there is growing interest in the role of navigation-assisted techniques in addressing these limitations, offering surgeons real-time intraoperative guidance to enhance precision and reproducibility.

This review examines the impact of navigation on surgical precision, clinical outcomes, and complication rates while also addressing limitations and prospects.

## 2. Navigation Principles

**Figure 2:**
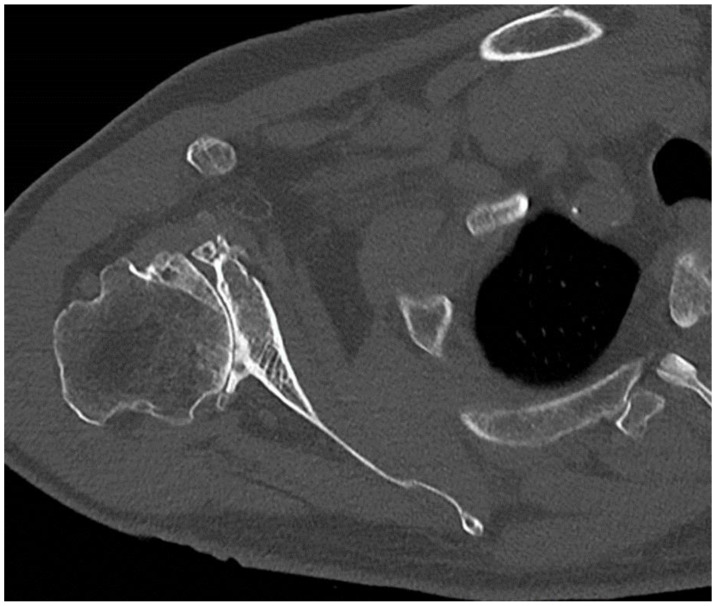
CT scan of the shoulder showing bone deformity due to osteoarthritis.

Numerous companies focusing on prosthetic devices have crafted proprietary software solutions designed to aid in the preoperative planning of shoulder arthroplasty. All the mentioned systems utilize preoperative 3D computed tomography (CT) to produce a three-dimensional model of the scapula, allowing surgeons to virtually position the glenoid component within this model before the surgery begins (Figure 2).

This approach guarantees precise visualization of glenoid version, inclination, and implant positioning, enabling surgeons to assess the need for any augmentations. Experts widely agree on the value of these three-dimensional planning software tools, with evidence suggesting they improve implant positioning without leading to high costs or added radiation exposure [20,21].

## 3. When to Navigate

Post-traumatic deformities following fractures present additional challenges in shoulder arthroplasty. Malunited fractures, bone loss, and altered joint morphology can make implant positioning more difficult and increase the risk of complications [22,23]. Navigation systems can assist in these complex cases by providing the real-time visualization of altered bony anatomy and facilitating accurate implant alignment [11,24]. This approach may help in restoring biomechanics more effectively, particularly in patients with severe post-traumatic changes [25].

The improper positioning of glenoid and humeral components can precipitate complications such as implant loosening, joint instability, and scapular notching, significantly impacting long-term surgical success. The intricacies of anatomical distortion in advanced OA further complicate traditional placement techniques. The integration of computer-assisted navigation in shoulder arthroplasty aims to mitigate these challenges by improving intraoperative accuracy and reproducibility [26].

## 4. Preoperative Time: CT Scan and Planning

**Figure 3:**
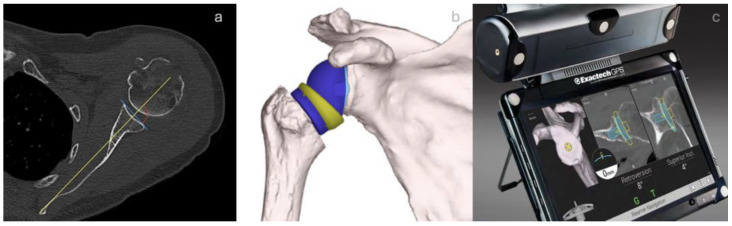
The surgical procedure for reverse shoulder arthroplasty with GPS navigation involves a pre-operative CT scan (**a**), planning (**b**), and intraoperative navigation (**c**).

Once the clinical evaluation is complete and the choice is made to perform a shoulder arthroplasty, if the surgeon opts for a navigated solution, the patient will be asked to perform a high-resolution CT. Once the CT is performed, it facilitates the generation of a detailed surgical blueprint, allowing surgeons to preoperatively optimize implant positioning (Figure 3).

The surgeon can select their preferred navigation software to accurately position the glenoid component within the three-dimensional rendering of the patient’s scapula before surgery begins. This software provides clear visuals for the version, inclination, and placement of the glenoid implant, which helps evaluate possible enhancements to address inclination and version issues.

Additionally, it enables the measurement of the lateralization and elongation of the humeral stem relative to the acromion and the original glenoid rotation center, facilitating a comparative analysis with the proposed implant’s modifications. Patients with post-traumatic deformities often present significant anatomical challenges that complicate prosthetic placement. In cases of prior fractures with malunion or bone loss, navigation allows surgeons to more accurately restore joint congruity. By offering improved visualization of the modified anatomy, navigation systems enhance the capacity to position implants in a biomechanically sound manner. This advancement enables the assessment of augment utilization, which may decrease the likelihood of loosening and enhance long-term outcomes [27]. Furthermore, the dimensions of the glenosphere and the insert’s lateralization and size, along with the range of motion, can be assessed to better understand the humerus’s movement relative to the scapula during adduction, abduction, internal and external rotation, anterior elevation, and retro position of the implant.

The systems can effectively pinpoint the contact area between the humeral implant and the scapula by demonstrating the motion range of the suggested implant. Additionally, they highlight the essential point needed to adjust component positioning, making it easier to implement changes for optimal joint recovery.

## 5. Intraoperative Navigation

At present, there exists only one GPS-based system that facilitates intraoperative navigation for the placement of the glenoid component, specifically the ExactechGPS Shoulder Application (Exactech, Gainesville, FL, USA) [28]. Similar to the technologies employed in hip and knee arthroplasty, the GPS system relies on a stable point on the patient’s anatomy in conjunction with the surgeon’s input to produce a stereotactic map. All instruments are referenced to this three-dimensional (3D) map, which is synchronized with the preoperative plan to ensure precise placement at the intended location [29]. It employs real-time tracking technologies, including optical and electromagnetic guidance, to enhance component placement accuracy during surgery [30]. The GPS technology relies on a specific point in the patient’s anatomy; for shoulder navigation, this is a tracker secured to the coracoid. This system is further refined by inputs from the surgeon via an electronic handpiece, which generates a bone map for accurate component positioning along the Friedmann axis.

The navigation system supports every phase of the procedure, including reaming, drilling for the central cage or screw, and inserting and rotating the baseplate. Consequently, the positioning of the glenoid baseplate, as well as the central and peripheral screws, can be visualized in real time. This visualization is improved by precise feedback on their trajectory displayed in coronal and axial CT slices, compared to the path detailed in the preoperative plan [31].

Achieving correct glenoid component positioning is crucial for implant longevity and joint stability. Studies indicate that navigation-assisted techniques significantly enhance precision in version and inclination angles, reducing the incidence of implant loosening and biomechanical dysfunction [32]. Advanced modeling tools are also being developed to predict optimal glenoid fixation, reducing variability in patient outcomes [33]. While humeral component placement is less technically challenging than glenoid positioning, achieving proper retroversion and alignment remains essential for optimal biomechanics. Navigation-guided approaches facilitate superior intraoperative control, particularly in cases involving anatomical deformities or severe degenerative changes. Regrettably, to date, no navigation system possesses the capability to navigate the humeral version. Nonetheless, such systems generally facilitate the simulation of shoulder mobility in the context of preoperative planning, depending on various positioning scenarios.

Larose et al. conducted an extensive review of over 16,000 shoulder arthroplasties, showing a high level of intraoperative accuracy and precision in following the preoperative plan. When the planned positioning of the glenoid implant was compared to the actual intraoperative positioning, the findings revealed minimal discrepancies, specifically, a version of 0.6° ± 1.96°, an inclination of 0.2° ± 2.04°, and a distance from the initial starting point measuring 1.90 ± 1.21 mm [34].

In 2021, Hones et al. conducted a study comparing 200 RSA, 100 conventional and 100 navigated. The results of this study showed that the total number of screws used in conventional RSAs was 414, while only 344 were implanted in the navigated group, thus proving that navigation leads to the implantation of significantly fewer screws per patient (3.4 vs. 4.1, *p* < 0.001). Furthermore, this study showed how navigation leads to implanting significantly longer screws, with an average length of 35.0 mm for navigated RSA vs. 32.6 mm for conventional RSA (*p* < 0.001) [35].

Similar results were reported by Sprowls et al., who reported the implant of longer screws in navigated RSA when compared to conventional RSA (36.7 mm vs. 30 mm, *p* < 0.0001) and the use of fewer screws 2.5 ± 0.7 in navigated RSA compared to 2.8 ± 1 in conventional RSA *(p* = 0.047). Furthermore, this study demonstrated the improved awareness of glenoid bone loss, which resulted in a dramatic increase in augmented baseplates, 76.5% in the navigation group vs. 19,1% in the conventional group (*p* < 0.0001) [36].

Moreover, navigation-assisted arthroplasty has been correlated with a reduction in the rates of complications, including scapular notching, malalignment, and eccentric polyethylene wear. These factors collectively enhance implant longevity and minimize the necessity for revision surgeries [20].

A notable recognition should be extended to works such as the one authored by Giorgini et al., wherein the reader is presented with a comprehensive examination of preoperative planning, followed by an overview of the surgical technique, and subsequently, the outcomes pertaining to surgical duration (92  ±  12 min), inclination, and version, which are detailed both preoperatively and postoperatively. The average screw length documented was 33.5  ±  4.2 mm. Most importantly, this publication indicates that there were no reported failures of the GPS system, in addition to providing valuable insights and recommendations for surgeons aspiring to enhance their proficiency in utilizing this essential tool [37].

## 6. Outcomes

**Figure 4:**
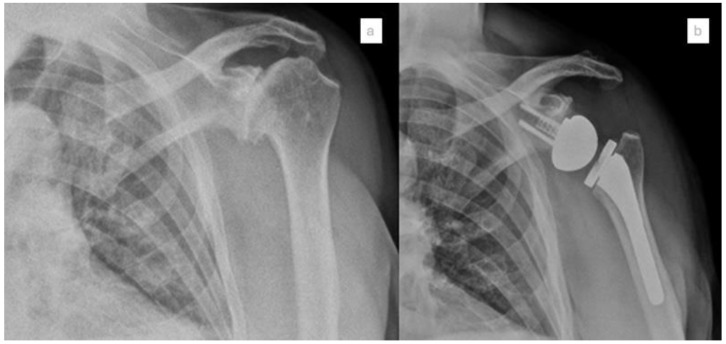
Pre-operative (**a**) and post-operative (**b**) X-ray of shoulder arthroplasty performed with GPS navigation.

Numerous early clinical studies have demonstrated a correlation between navigation and favorable outcomes, as well as reduced rates of complications (Figure 4).

Youderian et al. conducted a study that evaluated the clinical outcomes of navigated RSA in comparison to a cohort of conventional RSA, matched by sex and age. The findings indicated that patients in the navigation group exhibited enhanced internal and external rotation, a greater maximum lifting weight, and superior outcomes in the Simple Shoulder Test, Constant Score, and Shoulder Arthroplasty Smart compared to the conventional cohort. Furthermore, the navigated RSA group demonstrated an absolute risk reduction of 1.7% in postoperative complications and 0.7% in dislocations. However, no statistically significant differences were observed regarding glenoid implant loosening, acromial stress fracture, scapular notching, or revisions [31].

Holzgrefe et al. conducted a comprehensive evaluation of the clinical outcomes associated with navigated RSA in comparison to a cohort receiving conventional RSA [21]. The findings revealed a statistically relevant improvement in the navigated cohort regarding active forward elevation (135° for the navigated group versus 129° for the conventional group), active external rotation (39° for the navigated cohort compared to 32° for the conventional cohort), and Constant scores (71.1 for the navigated group versus 65.5 for the conventional group). Additionally, the study reported lower rates of complications (1.8% for the navigated cohort versus 5.3% for the conventional cohort), scapular notching (3.1% for the navigated group compared to 8.0% for the conventional group), and revision surgeries (0.9% for the navigated group versus 3.5% for the conventional group); however, it is important to note that these results did not reach the criteria for statistical significance.

In 2024, Andriollo et al. conducted a study that reported their surgical experience utilizing the Exactech GPS system and evaluated the outcomes in a cohort of 56 patients who underwent a surgical procedure for the implantation of RSA [20]. They documented various outcomes, including surgical duration (102 min, SD 16), baseplate and glenosphere sizes (ranging from 38 to 42), the number of screws used (2–3), screw length (32.9 mm, SD 5.7), and functional scores assessed by the quickDASH and Constant score metrics, along with pain scores measured by the Visual Analog Scale (VAS). These findings were consistent with the extant literature. The significance of this study lies in its status as the first manuscript to report a statistically significant increase in post-operative anterior elevation following navigated RSA.

Although the existing literature regarding the clinical outcomes associated with intraoperative navigation is somewhat limited, preliminary reports show promise and indicate that intraoperative navigation may enhance clinical outcomes for patients while potentially reducing complications [38]. Nonetheless, comprehensive long-term outcome studies are necessary to substantiate the clinical advantages of navigation.

## 7. Complications

Surgeons deciding to perform a navigated shoulder arthroplasty should keep in mind that even if it is a valuable tool, it is not free from risks.

In 2023, Larose et al. conducted research that reported an incidence of coracoid fractures of 0.05% (9 out of 16,723), with seven cases occurring intraoperatively.

Numerous studies have recorded infrequent cases of intraoperative tracker loosening or coracoid fractures occurring during the placement of trackers, conditions that are observed with higher frequency in patients exhibiting osteopenia [34].

The same complications were reported by Nashikkar et al., who reported a case of coracoid fracture and one case of tracker loosening in a cohort of 33 navigated shoulder arthroplasties [39].

In the same year, Youderian et al. conducted a study involving a cohort of over 16,000 shoulder arthroplasties. For TSA, no statistically significant differences were observed between the navigated and non-navigated cohorts with respect to postoperative complications, glenoid implant loosening, or revision rates. Furthermore, no substantial differences were noted in any of the TSA outcome metrics, apart from a greater degree of internal and external rotation observed in the navigated cohort. In the case of RSA, the navigated cohort demonstrated an absolute risk reduction of 1.7% concerning postoperative complications and a reduction of 0.7% in dislocations [31].

In their investigation, Tarallo et al. presented the outcomes of 46 patients who underwent RSA utilizing the Exactech GPS navigation system. According to their findings, intraoperative complications were notably distinct, comprising two coracoid fractures and one failure of the GPS system, which hindered the application of navigational assistance during the implantation of the glenoid component. In the months after the arthroplasty procedure, there were two instances of traumatic dislocations of the prosthetic implant, necessitating prosthetic revisions for both affected patients. Additionally, two cases of prosthetic infections emerged, each occurring within three months post-operation, which required surgical intervention for cleaning and replacement of the mobile components. Notably, one of the two infection cases necessitated further revision that involved the removal of the infected prosthesis and its replacement with an antibiotic-loaded spacer due to the recurrence of infection-related signs and symptoms [40].

A notable mention regarding the management of complications is warranted in reference to the study by Tarallo et al., which describes a rare instance of navigated RSA complicated by periprosthetic joint infection (PJI) occurring after six months, followed by a two-stage revision procedure. In the initial phase, the implant was extracted, and the screw and cage apertures were filled with morselized bone to emulate natural bone and restore bone stock. Upon resolution of the PJI, the second phase of the revision was executed using Exactech GPS, which, to the best of the authors’ knowledge, represents the first documented case of its kind [41].

## 8. Limitations

Despite its advantages, the adoption of navigation in shoulder arthroplasty presents several challenges, namely a longer surgical time, the learning curve of the surgeon, higher costs compared to conventional surgery, and some technical limitations.

The additional steps required for the integration of navigation systems may prolong surgical durations, potentially influencing workflow efficiency [42]. The effective utilization of navigation systems necessitates specialized training, which may temporarily affect procedural proficiency. To expedite the learning curve for novice users, virtual reality (VR)-based simulation training modules are currently under development [43]. The considerable costs associated with navigation technology, encompassing equipment, software, and maintenance, present obstacles to widespread implementation. Nevertheless, cost–benefit analyses indicate that enhancements in implant longevity and diminished revision rates may ultimately counterbalance these expenses over time [34]. Potential inaccuracies in tracking, calibration errors, and system malfunctions can jeopardize intraoperative reliability. Future advancements in sensor technology and AI-driven corrective algorithms may serve to mitigate these challenges.

## 9. Future Perspectives

Advancements in artificial intelligence (AI), augmented reality (AR), and robotic-assisted surgery are anticipated to further refine navigation in shoulder arthroplasty. AI-driven analytics may facilitate more precise preoperative planning, while AR-enhanced visualization tools could improve intraoperative guidance [44].

An emerging tool is mixed reality (MR), which aids shoulder arthroplasty through 3D planning. Surgeons wear MR glasses to view holograms of patient anatomy, thus enhancing component placement. Recently, MR received regulatory approval for glenoid wire navigation. However, evidence of MR’s effectiveness is limited; initial studies found no significant differences in guidewire positioning or operative time compared to traditional methods, and the cost vs. benefit of MR remains unclear [45].

## 10. Conclusions

Computer-assisted navigation has emerged as a pivotal innovation in shoulder arthroplasty, offering enhanced precision and potentially reducing complication rates associated with implant mispositioning. The role of navigation in addressing post-traumatic deformities is also gaining recognition, providing solutions for complex reconstructive cases. Nevertheless, long-term outcomes and continued research are needed to establish evidence-based best practices and refine integration strategies for navigated shoulder arthroplasty.
